# Unveiling the Constraints of COSMO-SAC for PEG-Water Liquid–Liquid Equilibrium Prediction

**DOI:** 10.1021/acs.iecr.5c04482

**Published:** 2026-05-05

**Authors:** Edgar T. de Souza, Murilo L. Alcantara, Paula Bettio Staudt, João A. P. Coutinho, Rafael de P. Soares

**Affiliations:** † Virtual Laboratory for Properties Prediction (LVPP), Chemical Engineering Department, Federal University of Rio Grande do Sul, Rua Ramiro Barcelos, 2777, Porto Alegre, Rio Grande do Sul CEP 90035-007, Brazil; ‡ CICECO - Aveiro Institute of Materials, Chemistry Department, University of Aveiro, Aveiro 3810-193, Portugal

## Abstract

This work studies the constraints of the COSMO-SAC model for predicting the liquid–liquid equilibrium (LLE) of polyethylene glycol (PEG) and water mixtures. The research extends the COSMO-SAC methodology to include polymer end groups in the σ-profile construction and employs a global optimization approach for LLE prediction across the entire composition range. The influence of various parameters, including end groups, hydrogen bond energies, and volume effects, on the LLE prediction is analyzed. The results indicate that while the COSMO-SAC model can qualitatively predict the phase behavior of PEG-water systems, including, for the first time, the closed-loop diagram, significant deviations from experimental data are observed for the LCST. The results obtained suggest that further improvements are required in both the combinatorial and residual terms to enhance the predictive accuracy of COSMO-SAC for complex polymer–solvent systems.

## Graphical abstract

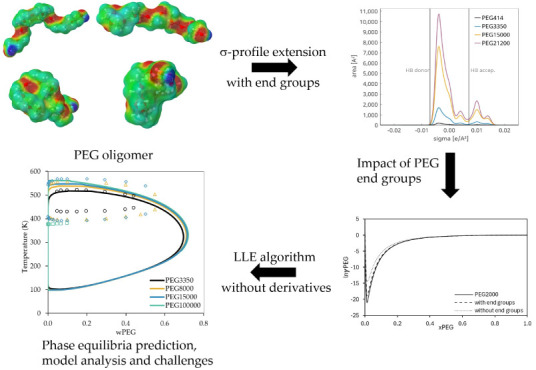

## 1 Introduction

Polymers are widely used in a range of applications due to their versatile and customizable material attributes. These properties can be modified by adjusting the chemical composition, molecular weight, or tacticity of the polymer, introducing diverse side chains, or incorporating other substances. A comprehensive understanding of the phase behavior of polymers is essential for processes such as synthesis, polymerization, and solvent separation from polymer solutions.^1,2^

Polyethers comprise a group of compounds with extensive industrial applications, including the formulation of cosmetics, pharmaceuticals, and lubricants.^3,4^ The research into aqueous-based lubricants presents an important area of interest, seeking to supplant conventional oil-based lubricants with more environmentally friendly alternatives. Consequently, the capacity to predict the solubility of polyethers in water under varying conditions (which can encompass a wide range of temperatures and pressures) is very relevant. This predictive capability rationalizes the selection of candidates for laboratory testing, enabling the assessment of systems with more favorable properties.

The liquid–liquid equilibria (LLE) extraction process using aqueous biphasic systems (ABS) is another noteworthy application that uses polyethers in water to extract and purify biomolecules and other relevant compounds.^5–7^ Nevertheless, despite the efforts made by numerous authors to comprehend the intermolecular mechanisms that govern the formation of ABS, this phenomenon remains elusive and not fully understood.^8,9^ Even the phase equilibria of simple ethers in water still represent a challenge to predictive models.^10^

Many efforts have been made to model the phase behaviors of polymer solutions, including polyethers in water. Staudt et al.^11^ predicted the vapor–liquid equilibria (VLE) for several polymer solutions using COSMO-SAC. The authors made an extension to polymers by constructing the σ-profile of a repeating unit, and the full polymer σ-profile is achieved by multiplying it using the number of basic units of the macromolecule. This procedure was necessary to address the high computational cost of performing the quantum mechanical (QM) calculations for large macromolecules. Besides this approximation, the authors achieved good qualitative results for VLE. However, they claimed that an improvement in the combinatorial contribution would probably be necessary for the representation of LLE data. Kuo et al.,^12^ applied the COSMO-SAC to predict VLE and LLE of polymer–solvent systems with Elbro et al.^13^ model as the combinatorial term to include free volume effects. The authors obtained comparable results, regarding the activity coefficient, to what was previously achieved using UNIFAC-FV,^14^ the group contribution model with the free volume (FV) contribution. The authors suggested that the use of different polymer chain configurations might improve the COSMO-SAC estimates. Recently, Silva et al.^15^ discussed the challenges of using COSMO-RS to describe polymer solution behavior. The authors assessed the influence of polymer conformation in predicting the activity coefficient and used the PEG/water system as an example. With the conformer chosen by molecular dynamics (MD) simulations, the authors could obtain results in qualitative agreement with the activity coefficients of PEG in water, obtained from SLE data. Nevertheless, the authors did not compare the predicted results with activity coefficients in higher temperatures, where the LLE happens. Additionally, at the highest molar mass tested (35 000 g/mol), the deviation of PEG activity coefficients from SLE data still spanned several orders of magnitude. Also, they were unable to calculate the LLE diagrams with COSMOThermX software, due to numerical limitations arising from the high-molecular-weight asymmetry between polymer and solvent. However, whenever such numerical issues arise, it remains unclear whether they are purely computational or reflect an intrinsic limitation of the model in predicting phase separation.^16,17^

Lindvig et al.^18^ emphasized the numerical issue, noting that conventional formulations often fail when the equilibrium polymer concentration becomes exceedingly low, potentially falling below the numerical precision limits of computers. The authors proposed robust stepwise-tracing algorithms, which typically rely on an initial instability search (locating spinodal points) and subsequent refinement using second-order methods. However, such approaches fundamentally require the derivatives of Gibbs energy, which are computationally difficult or impractical to obtain for continuum solvation models like COSMO-SAC.^19^ Given these numerical challenges, and since COSMO-SAC lacks analytical derivatives, a more robust computational method is necessary.

Regarding the SAFT models, various versions have been employed to model LLE of PEG + water system.^20–22^ However, they need binary interaction parameters or must be directly fitted to the LLE data. Another problem is the extension of its parameters to ternary mixtures like ABS, in which a completely new set of parameters would be needed. Most SAFT models used for aqueous mixtures of PEG also implemented temperature or molecular weight-dependent parameters to describe the experimental data. Most recently, Valsecchi et al.^23^ used SAFT-γ Mie to correlate the LLE of PEG and water. They could correlate the data without the need for temperature or weight-dependent parameters. Furthermore, by only using LLE data in the parameters fitting procedure they could also describe density, enthalpy of mixing, and VLE with the same set of parameters. However, the need for mixture data remains a problem in this approach to predict and investigate new polymer–water systems, as well as the high complexity of SAFT models.

Group contributions models have been widely applied to polymer–solvent systems.^24–26^ Tritopoulou et al.^26^ used the UNIFAC residual term coupled with the Elbro et al.^13^ combinatorial term. The authors achieved good qualitative results, but direct LLE fitting parameters had to be added. The authors also showed the importance of the end groups in the LLE description. Even though the number of PEG end groups is just two per molecule, much less than those of the chain groups, its presence affected phase behavior in PEG-water systems. The authors mentioned that not even qualitative correlation results could be achieved when the end groups were neglected. Thus, they added specific interaction parameters between the end group of PEG and its repeating unit, and between the end group and the water molecule. This is relevant, since in COSMO approaches the end groups are usually neglected.^11,12,27^

Although correlative models, such as UNIFAC and SAFT variants, can adequately correlate the phase equilibria between PEG and water, they are not useful for preselecting compounds that could form a ternary mixture in an ABS, for instance. In many applications, a predictive approach would be desirable. Moreover, all estimated parameters in these models must be recalibrated whenever a new compound is added to the system, requiring experimental data of this specific new mixture. Thus, efforts to understand the current limitations of COSMO-SAC and to define future directions for its enhancement remain worthwhile objectives.

In this work, an extended method to explicitly include end groups in σ-profile construction is presented. Furthermore, a global optimization approach is employed for the LLE prediction of PEG and water, providing a derivative-free strategy that circumvents the need for analytical Gibbs free energy derivatives, which are not readily available in COSMO-based activity coefficient models, while also addressing the numerical challenges reported^15^ for the PEG–water system arising from the pronounced molecular weight asymmetry between polymer and solvent, which leads to very low equilibrium mole fractions of PEG and strongly asymmetric activity coefficients. Additionally, the COSMO-SAC residual and combinatorial terms are analyzed, verifying the parameters’ influence in LLE. The aim is to address the current limitations of COSMO-SAC when predicting polymer–solvent systems, in particular the PEG-water mixture used in this study.

## 2 The COSMO-SAC Model

In COSMO-SAC, similarly to UNIFAC,^28^ the activity coefficient is defined as a sum of two contributions:

1 $$\ln\gamma_{i}=\ln\gamma_{i}^{comb}+\ln\gamma_{i}^{res}$$

where $\ln\gamma_{i}^{res}$ is the residual activity coefficient, and $\ln\gamma_{i}^{comb}$ is the combinatorial contribution.

The residual contribution comes from the pairwise surface interaction theory. First, it is necessary to compute the σ-surface, which is the three-dimensional cavity formed by the induced surface charge densities around the molecule when surrounded by a perfect conductor. This cavity consists of many segments of identical area *Q*_*eff*_. Each segment *m* has different apparent surface charge densities, *σ*_*m*_. For the calculations, the σ-profile is used, which is the distribution of these surface charge densities over the molecular cavity. Subsequently, for modeling a fluid or a mixture, a combination of pairwise interacting segments is considered. As described in Soares and Staudt,^29^ each pair of segments *m* and *n* constitutes two tangent molecules interacting with an associated pair formation energy *u*_*mn*_ and Boltzmann factor Ψ_*mn*_, calculated as

2 $$\Psi_{mn}=\exp\left(-\frac{u_{mn}}{kT}\right)$$

Each of those segments is considered to have an associated activity coefficient Γ_*m*_ given by

3 $$\Gamma_{m}={\left[\underset{n}{\sum }\Theta_{n}\Gamma_{n}\Psi_{mn}\right]}^{-1}$$

where the surface area fractions Θ_*m*_ of a segment type *m* in a mixture are computed as

4 $$\Theta_{m}=\frac{\underset{i}{\sum }x_{i}Q_{m}^{i}}{\underset{j}{\sum }x_{j}q^{j}}$$

where $q^{i}=\underset{n\in i}{\sum }Q_{n}^{i}$ is the total cavity area of molecule *i*. The residual contribution is then calculated as

5 $$\ln\gamma_{i}^{\mathrm{res}}=\underset{m\in i}{\sum }\nu_{m}^{i}\cdot \left(\ln\Gamma_{m}-\ln\Gamma_{m}^{i}\right)$$

where $\nu_{m}^{i}$ is the number of times the segment type *m* appears in molecule $i,\nu_{m}^{i}=Q_{m}^{i}/Q_{\mathrm{eff}};Q_{m}^{i}$ is the area of the segment type *m* in molecule *i*; $\Gamma_{m}^{i}$ is the activity coefficient of the segment *m* in the reference state of a pure fluid. This pure fluid activity coefficient is computed utilizing the same equations but evaluated as a pure compound.

Equation 3 is a system of equations, one for each segment type *m*, and it must be solved numerically. Furthermore, the COSMO-SAC variant used here^30^ can handle multiple hydrogen bond energies for different donor–acceptor pairs. This is feasible because each molecule surface segment retains the information about its corresponding atom in this implementation. This is accounted for in *u*_*mn*_ as follows:

6 $$u_{m,n}=\frac{\alpha^{\prime }}{2}{\left(\sigma_{m}+\sigma_{n}\right)}^{2}+c_{hb}\max\left\{0,\sigma_{acc}-\sigma_{hb}\right\}\min\left\{0,\sigma_{don}+\sigma_{hb}\right\}$$

and $\alpha^{\prime }=f_{pol}\times \left(0.3a_{eff}^{3/2}/\epsilon_{0}\right)$ ; $\epsilon_{0}$ is the electric permittivity in vacuum; *σ*_*acc*_ is the acceptor charge density, and *σ*_*don*_ is the donor charge density; *f*_*pol*_ is the polarizability factor, which fits the electronic polarization that occurs during contact between two segments; *c*_*hb*_ is the hydrogen bond constant; and *σ*_*hb*_ is the charge density threshold for interaction to occur. These last three variables are universal parameters. From here and forward, this variant will be called COSMO-SAC-HB2. In eq 6, the hydrogen-bond interaction constant *c*_*hb*_ is written in a compact form for clarity. In the COSMO-SAC-HB2 formulation, this term does not represent a single universal parameter. Instead, multiple hydrogen-bond constants are defined for different donor–acceptor pairs as described by Souza et al.^31^

The combinatorial contribution used in this work is the Elbro et al.^13^ equation:

7 $$\ln\gamma_{i}^{\mathrm{comb}}=\ln\frac{\Phi_{i}}{x_{i}}+1-\frac{\Phi_{i}}{x_{i}}$$

where $\Phi_{i}=x_{i}\left(\zeta v_{i}-r_{i}\right)/\underset{j}{\sum }x_{j}\left(\zeta v_{j}-r_{j}\right)$ is the volume fraction, which is adapted in this work to include a constant *ζ*. When *ζ* = 0, the model corresponds to the Flory–Huggins (FH)^32^ equation, and when *ζ* = 1, the volume fraction reverts to the original form described by Elbro et al.^13^ This volume constant *ζ* is used as a parameter to analyze the effect of volume in the equilibrium calculations; *r*_*i*_ is the molecular volume of compound *i* resulting from the COSMO calculation; *x*_*i*_ is the mole fraction of component *i*, and *v*_*i*_ is the molar volume of component *i*. The polymer molar volumes are calculated utilizing the Tait equation,^33^ and for the solvent, a polynomial correlation was created with NIST TDE^34^ experimental data.

The σ-profiles used in this work were computed using TURBOMOLE V7.4 2019,^35^ employing DFT with the BP-86 functional, using either the def_TZVP basis set or the def2_TZVPD basis set with the FINE marching tetrahedron cavity.^36^ All COSMO-SAC calculations were performed using the COSMO-SAC-HB2 model as implemented in JCOSMO software,^30^ freely available at https://www.ufrgs.br/lvpp/download/jcosmo/, utilizing either the BP-TZVP or the BP-TZVPD-FINE parametrizations reported by Souza et al.^31^ It is important to note that all calculations were performed using a single conformer. Additionally, unless explicitly stated otherwise, the linear PEG conformation is the default PEG geometry adopted.

## 3 Polymer Extension with End Groups

Strategies for generating polymer σ-profiles using oligomer models have been well established in the literature.^11,12,37,38^ In these approaches, oligomers are typically capped with generic groups, such as methyl groups, whose primary purpose is to saturate chemical bonds and imitate the continuation of the polymer chain. As a result, the σ-profile is assumed to reflect primarily the properties of the repeating unit. This assumption is generally considered valid for high-molar-mass polymers, for which terminal groups are commonly neglected.^11,15,27^ However, when end groups differ in chemical nature from the polymer backbone, their specific thermodynamic contribution can become non-negligible. This situation is particularly relevant to PEG, whose structure begins and ends with polar hydroxyl groups that contrast with the ether backbone and can significantly affect practical applications.^39,40^ Moreover, in COSMO-based studies PEG is often represented using one or two methyl groups as terminations.^27,41,42^ Since the present work introduces a method that enables the explicit inclusion of PEG terminal groups, it is important to highlight this recurring simplification in the literature, which should be carefully addressed in future studies.

In response to this, the methodology of Staudt et al.^11^ was adapted in this work to explicitly include the end groups of polymers. The initial step is the identification of a repeating unit. Once this unit is defined, several oligomers are created for PEG, all containing an odd number of repeating units. Subsequently, all oligomer structures are optimized using TURBOMOLE, and their surface charges are calculated as for usual molecules.

After determining the apparent surface charge of the entire molecule, only the surface charge of the central repeating unit of the oligomer, along with the end groups, are retained. This is performed by creating a custom file, which assigns atomic weights to selected atoms of the oligomer. In this procedure, atoms belonging to the terminal groups are assigned unit weight, while atoms belonging to the repeating unit are assigned a weight equal to the number of basic units *N*_*b*_, necessary to reach the average molecular weight of the polymer *MW*_*p*_:

8 $$N_{b}=\frac{MW_{p}-\sum MW_{e}}{MW_{r}}$$

where *MW*_*e*_ is the molecular weight of an end group and *MW*_*r*_ is the molecular weight of the repeating unit. Figure 1 shows an example of the obtained σ-surface for PEG.

**Figure 1:**
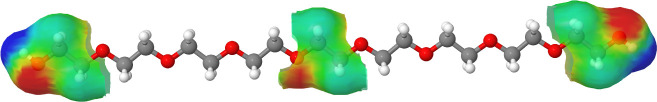
Surface charge of the central unit, and end groups for PEG.

The repeating unit area and volume parameters used in the calculations are determined from the difference in total volume and area of the two largest consecutive oligomers. The end groups’ total volume and area are obtained by subtracting the repeating unit parameters from the entire oligomer, according to its number of repeating units. The instructions for constructing custom files compatible with JCOSMO are available at https://lvpp.github.io/jcosmo-docs/polymer/. An illustrative example of a JCOSMO.custom file used in this work is presented in Figure S1 of the Supporting Information for full reproducibility. For more details on the methodology, including when dealing with copolymers, please refer to the work of Staudt et al.^11^

## 4 LLE Solver Algorithm

As previously discussed, the calculation of LLE in polymer solutions involves substantial numerical challenges that frequently lead to the failure of classical algorithms. These difficulties mainly arise from the pronounced molecular weight asymmetry between the polymer and the solvent.^15,18^ Existing approaches that overcome such issues typically rely on model derivatives, which are not as straightforward for COSMO-based models as for classical activity models. Therefore, an alternative derivative-free algorithm is proposed.

A similar approach to that propounded by Staudt and Soares^43^ is presented here to solve LLE and obtain the equilibrium diagram for polymer–solvent systems. First, the minimization of the overall system Gibbs free energy is realized utilizing the DIviding RECTangles (DIRECT) global optimization algorithm:^44^

9 $$\frac{G\left(n\right)}{RT}=\overset{N_{p}}{\underset{k=1}{\sum }}\overset{N_{c}}{\underset{i=1}{\sum }}n_{i}^{k}\ln f_{i}^{k}$$

10 $$n_{i}^{1}=\beta_{i}^{1}n_{i}$$

11 $$n_{i}^{k}=\beta_{i}^{k}\left(n_{i}-\overset{k-1}{\underset{j=1}{\sum }}n_{i}^{j}\right)\;i=1,2,...,N_{c}\;k=2,...,N_{p}-1$$

where *n*_*i*_ is the overall number of moles of the component *i*; $f_{i}^{k}$ and $n_{i}^{k}$ are the fugacity and number of moles of the component *i* in the phase *k*, respectively; *N*_*p*_ is the maximum number of phases at equilibrium, which is considered to be known; *N*_*c*_ is the number of components and $\beta_{i}^{k}$ are the decision variables, bounded between 0 and 1. This optimization problem shown in eq 9, utilizing the decision variables $\beta_{i}^{k}$ , is a bound-constrained problem.

Subsequently, the best result achieved by DIRECT algorithm is used as the initial guess in a classical Rachford-Rice flash solver,^45^ which is adapted to handle mass-based activity coefficients and mass fractions, according to their definitions:^46,47^

12 $$\ln a_{i}=\ln\left(x_{i}\gamma_{i}\right)=\ln x_{i}+\ln\gamma_{i}$$

13 $$\ln a_{i}=\ln\left(w_{i}\gamma_{i}^{\prime }\right)=\ln w_{i}+\ln\gamma_{i}^{\prime }$$

14 $$\gamma_{i}^{\prime }=\frac{\gamma_{i}}{M_{i}\left(\overset{K}{\underset{j=1}{\sum }}w_{j}/M_{j}\right)}$$

where $\gamma_{i}^{\prime }$ is the mass-based activity coefficient, and *a*_*i*_ is the activity of component *i*. It should be noted that the DIRECT algorithm provides this initial guess operating on a molar basis, which is subsequently converted into mass basis for use in the Rachford-Rice flash solver. This procedure was found to be sufficient for the cases studied in this work.

The molar compositions are then converted to mass fractions and the flash solver receives this information from the two phases. The global composition, which is the average of both mass fractions, and the temperature at which we want to find the equilibrium are other inputs in the algorithm. The convergence criterion is reached when the difference in the phase split ratio between the liquid phases of the current and previous iterations is lower than 10^–5^. It is worth noting that the partition coefficients *K*_*i*_ used in the flash solver must be calculated using the logarithm of $\gamma_{i}^{\prime }$ , to avoid errors caused by the very low *γ*_*i*_ values that are usually found, particularly with molar mass increase (for instance, lower than 10^–402^ at 100,000 g·mol^–1^ and 0.36 PEG mass fraction).

## 5 Results

### 5.1 Influence of End Groups in Activity Coefficient

The influence of the end groups on the activity coefficient was addressed by comparing the obtained result with those of the full molecule, the monomer multiplied by considering the end groups, and the repeating units alone. Due to the limitations of computing quantum mechanics for very large molecules, owing to the high computational cost, the ChemDraw 3D software was used to perform an optimization using the MM2 force field for this test. With the obtained structure, the QM calculations were carried out without any further structure optimization, allowing the calculation for the entire molecule. The monomers were also calculated in this manner for this specific test for the sake of comparison, using the def-TZVP quantum-chemical level, while the FH combinatorial term was considered. Figure 2 shows the results for PEG with 2000 g/mol. Without considering the end groups, the activity coefficient is overestimated. The correlation coefficient *R* ^2^ between the activity coefficient logarithm of the full molecule of PEG and the multiplied monomer with the end groups is 0.9919, while when neglecting end groups, it falls to 0.6658. This shows the importance of considering the end groups, which agrees with what was observed by Tritopoulou, et al.^26^ when applying UNIFAC to this system. We could not evaluate the results for PEG full molecule at higher molar weight, due to the limitations of the ChemDraw 3D software.

**Figure 2:**
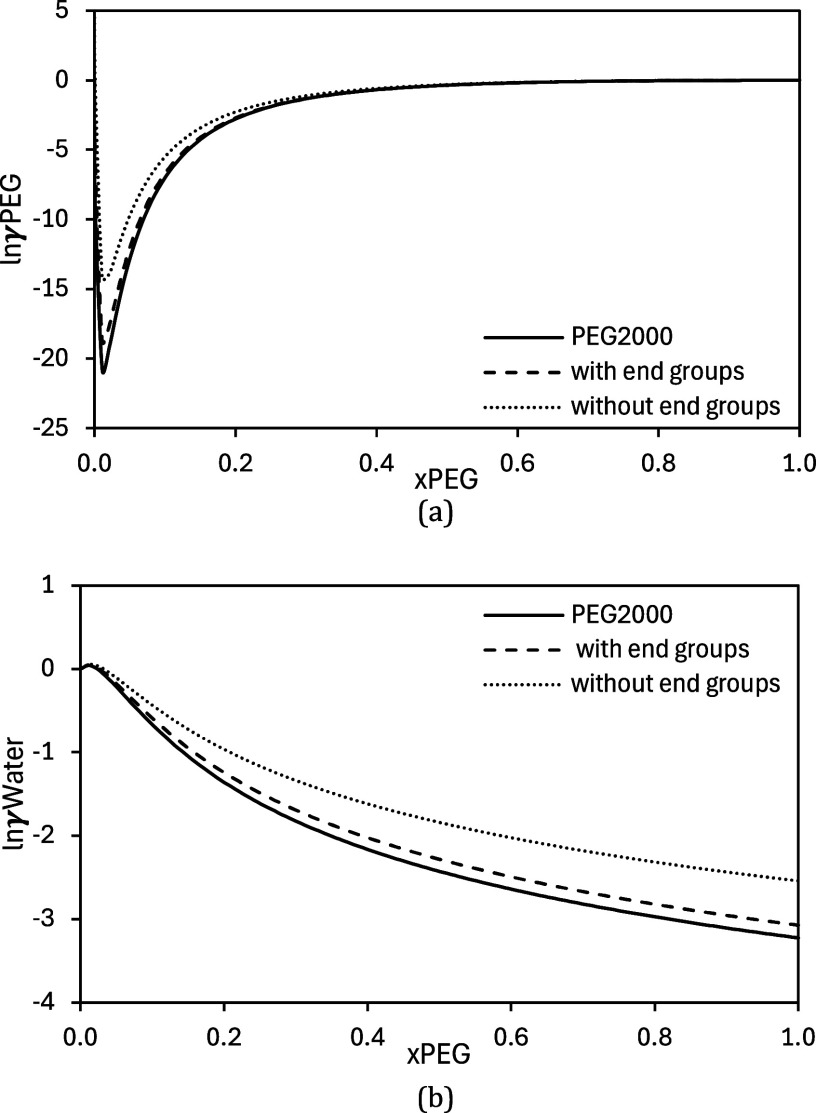
Influence of end groups on the activity coefficients at 298 K of (a) PEG2000 and (b) water. The solid line represents calculations using the full PEG molecule, while the dashed and dotted lines represent predictions based on monomer multiplication, with and without terminal hydroxyl groups, respectively.

Table 1 presents the Average Absolute Relative Deviation (AARD) and Average Absolute Deviation (AAD) values for different PEG molar masses, comparing the logarithm of activity coefficients calculated with end groups (lnγ_i_,_we_) and without end groups (lnγ_i_,_wo_) at 298 K, using 110 composition points evenly distributed over the entire PEG mass fraction range. As the molar mass increases, the AARD tends toward zero, whereas the AAD for lnγ_PEG_ remains nearly constant. The decrease in AARD is associated with the substantial increase in the magnitude of lnγ_PEG_, which reduces the relative deviation while nearly the same absolute difference between the two calculations is obtained. For water, both AARD and AAD approach zero with increasing molar mass, indicating that the influence of the terminal groups becomes progressively less significant for lnγ_Water_.

**Table 1:** Average Absolute Deviation (AAD) and Average Absolute Relative Deviation (AARD) between Activity Coefficients Calculated with End Groups (lnγ__i_,we_) and without End Groups (lnγ_i_,_wo_) for Different PEG Molar Masses^a^

Molar weight (g mol^–1^)	AARD lnγ_PEG_	AARD lnγ_Water_	AAD lnγ_PEG_	AAD lnγ_Water_
2,000	211.73%	39.12%	3.88	7.08 × 10^–02^
15,000	5.64%	5.20%	3.81	1.15 × 10^–02^
100,000	0.74%	1.53%	3.92	1.83 × 10^–03^
1,000,000	0.07%	0.94%	3.94	1.85 × 10^–04^

a The deviations are defined as $\mathrm{AAD}=\frac{1}{N}\overset{N}{\underset{k=1}{\sum }}\left|\ln\gamma_{i,we}^{\left(k\right)}-\ln\gamma_{i,wo}^{\left(k\right)}\right|$ and $\mathrm{AARD}\left(\%\right)=\frac{100}{N}\overset{N}{\underset{k=1}{\sum }}\left|\frac{\ln\gamma_{i,we}^{\left(k\right)}-\ln\gamma_{i,wo}^{\left(k\right)}}{\ln\gamma_{i,we}^{\left(k\right)}}\right|$ Where *N* is the total number of points and *k* denotes each calculated point.

Figure 3 shows the composition dependence of the AAD for PEG with a molar mass of 1,000,000 g·mol^–1^. The left axis corresponds to the AAD of PEG, and the right axis to the AAD of water. In the water-rich region, the highest AAD values for lnγ_PEG_ are observed, indicating greater sensitivity to the presence of terminal groups. As the PEG mass fraction increases, the influence of the terminal groups on lnγ_PEG_ decreases. This behavior can be explained by the higher probability of interactions between water molecules and the polymer end groups in the water-rich region, which enhances their effect on lnγ_PEG_. For water, the AAD increases as the PEG composition increases. However, its magnitude remains several orders of magnitude lower than that observed for lnγ_PEG_. Even at high PEG mass fractions, the deviations in lnγ_Water_ are on the order of 10^–4^–10^–3^, indicating that the influence of terminal groups on the water activity coefficient is minor in high molar mass.

**Figure 3:**
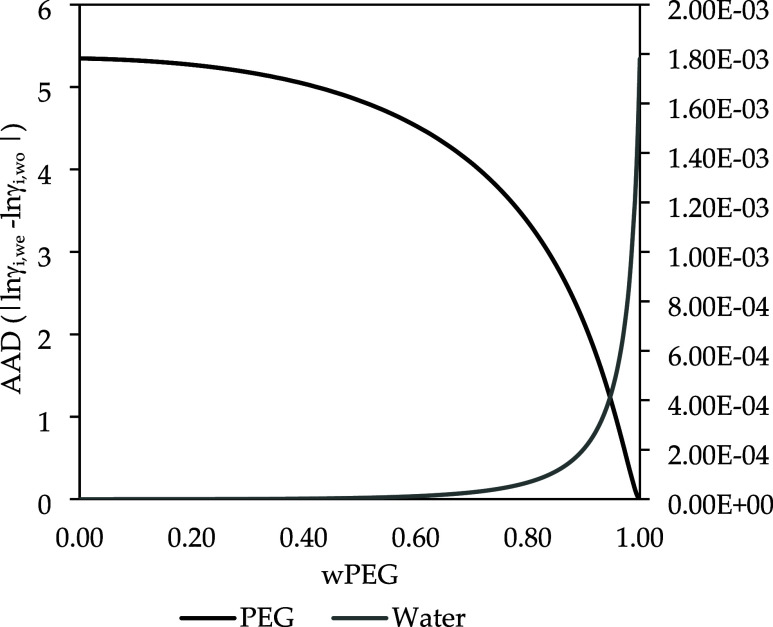
Composition dependence of the Average Absolute Deviation (AAD) between activity coefficients calculated with (lnγ_i_,_we_) and without (lnγ_i_,_wo_) end groups for PEG with molar mass of 1,000,000 g·mol^–1^ at 298 K. The left axis corresponds to AAD of lnγ_PEG_ and the right axis represents AAD of lnγ_Water_.

Authors usually do not consider the end groups in the COSMO computations, as in the works of Loschen and Klamt,^27^ Staudt et al.^11^ and Silva et al.^15^ Figure 4 illustrates the impact of considering the end groups on the LLE of PEG 2000 g·mol^–1^ and water. It can be observed that with the end groups, we achieve a higher mass fraction of water, and the curve shrinks. Using only the monomer groups leads to an increase in the upper critical solution temperature (UCST), but with a lower mass composition of water. The end groups introduce only a minor change on the LCST predictions, with the compounds miscible in all proportions only below 100 K. It is possible to observe that the inclusion of the hydroxyl end groups affected way more the UCST, indicating that it could be more sensitive to the residual contribution, since the combinatorial term would not be much changed with this addition to the molecule. The inclusion of the end groups does not match perfectly the LLE diagram generated with the full PEG2000 molecule, but it is a better approximation to optimize computational cost.

**Figure 4:**
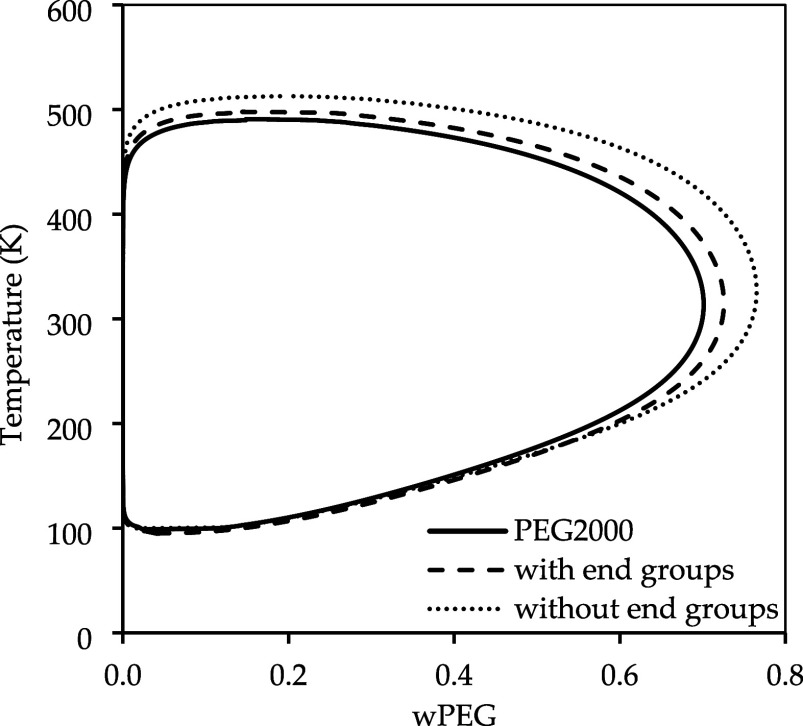
Influence of the inclusion of the end groups in LLE of PEG2000 and water.

It is important to stress that such end groups importance is not expected to be general for polymer–solvent systems. In most polymers, end groups are chemically similar to the repeating units and therefore exhibit comparable σ-profiles and interaction strengths. Under those conditions, their contribution scales approximately with their surface fraction and becomes negligible at high molar mass. The pronounced effect observed here is thus a specific feature of the PEG–water system, arising from the strong hydrogen-bonding contrast between hydroxyl termini, ether backbone segments, and water.

### 5.2 Parametrization Impact on the LLE

The influence of using BP-TZVP or BP-TZVPD-FINE in the computation of LLE is shown in Figure 5. The results with the FINE method led to an even richer PEG phase and lower LCST, increasing the deviations from experimental data. This result indicates that the FINE method may not be suitable for this case.

**Figure 5:**
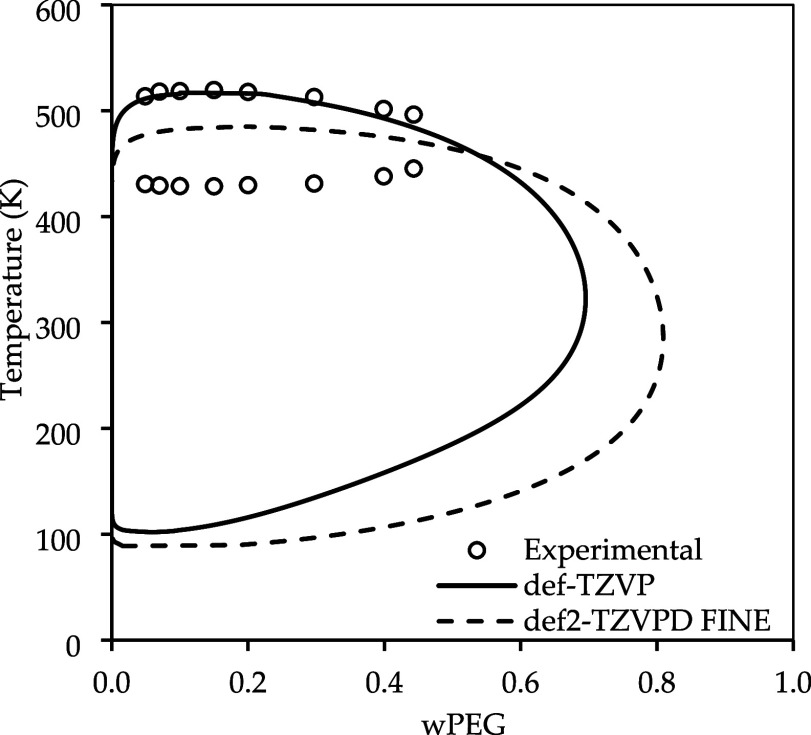
Impact of the parametrization used in the LLE calculation of PEG 3350 g·mol^–1^ and water. Experimental data taken from Bae et al.^50^.

The poorer performance observed for BP-TZVPD-FINE compared to BP-TZVP can be rationalized by considering the intrinsic sensitivity of COSMO-SAC to subtle changes in the σ-profile description. Although BP-TZVPD-FINE employs a larger basis set with diffuse functions and a finer cavity discretization, previous systematic assessments comparing BP-TZVP and BP-TZVPD-FINE have shown that the latter does not necessarily improve LLE predictions and may even deteriorate them.^48,49^ The FINE cavity generates a narrower and more resolved segment charge distribution; however, the COSMO-SAC relies on a delicate balance between residual and combinatorial contributions. Small modifications in the σ-profile may therefore disturb this balance, reducing favorable error compensation that may be present in the BP-TZVP. From this point forward, the BP-TZVP is adopted for all subsequent LLE calculations.

### 5.3 Hydrogen Bond Energy Influence in LLE

As discussed previously, our COSMO-SAC implementation can handle multiple hydrogen bond energies for different donor–acceptor pairs. Figure 6 shows the influence of the ether-water HB energy on the LLE. As expected, the hydrogen bond energy has a strong impact on the equilibrium. As the ether-water HB energy increases, the PEG mass composition decreases, and more water is soluble in the polymer, and the UCST is significantly affected, decreasing with increasing HB. On the other hand, the LCST increases with HB but is much less impacted than the UCST. This could indicate that enthalpic interactions have a higher impact on the UCST than on the LCST. A feasible explanation for the decrease of the UCST is that with a stronger hydrogen bond, the system has a more negative Δ*H*_*mix*_, thus mixing becomes more favorable even at lower temperatures.

**Figure 6:**
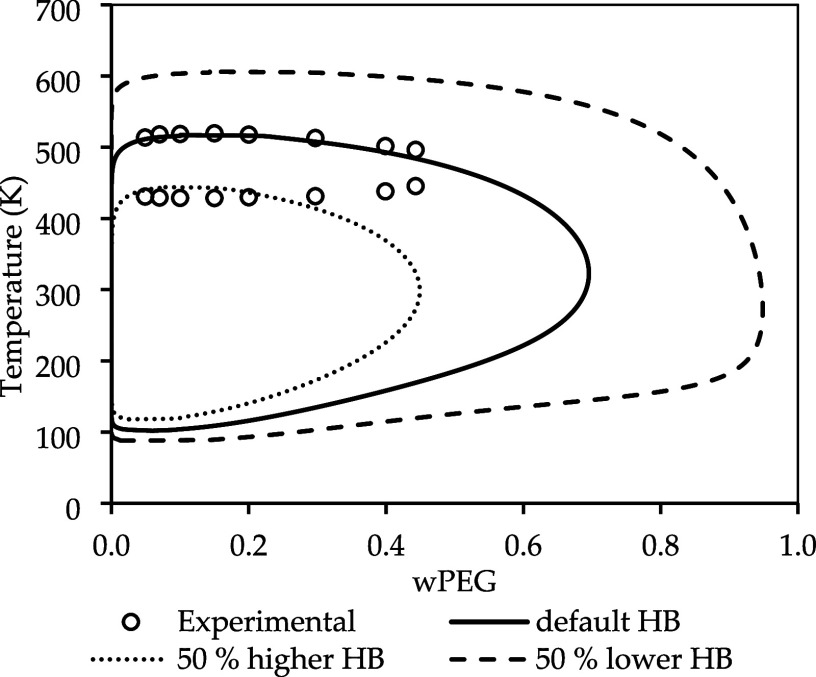
Ether-water hydrogen bond influence in LLE of PEG (3350 g·mol^–1^) and water. Experimental data taken from Bae et al.^50^.

One possible explanation for the deviations observed is that the COSMO-SAC residual term does not adequately describe the enthalpy effects, suggesting that improvements are needed for the residual term. Figure 7 shows the predicted enthalpy of mixing using FH for the PEG3000/water system, along with the experimental data from Malcom and Rowlinson^51^ at 353.45 K. The predicted enthalpy is underestimated for mass compositions of PEG below 0.8 and overestimated above this composition range. It is also noteworthy that our model predicts an LLE at this temperature, resulting in a linear behavior of enthalpy in the composition range below 0.8, which makes it right-skewed. However, it predicts values that are qualitatively correct; furthermore, if we increase the hydrogen bond between ether and water, we can improve the results as shown in Figure 7. Additionally, we also tested hydrogen bond parameters with temperature dependence, but no improvement in LLE prediction was observed.

**Figure 7:**
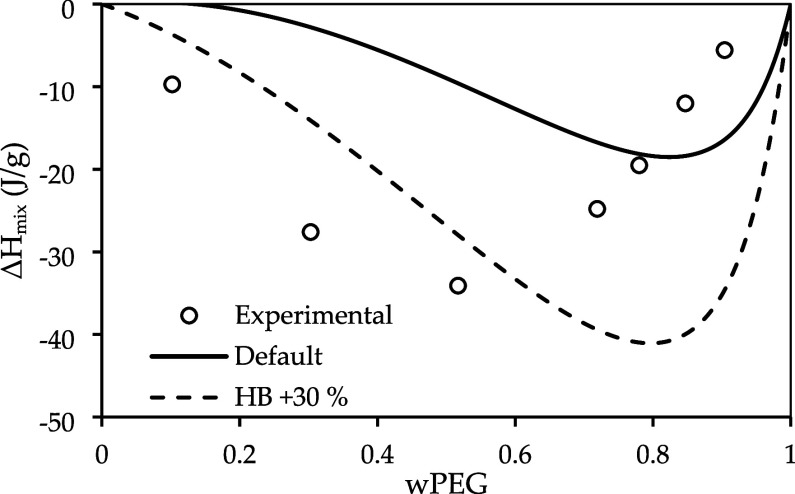
Predicted mixing enthalpy of PEG (3000 g·mol^–1^) and water at 353.45 K, compared with experimental data from Malcolm and Rowlinson.^51^

While adjusting the HB energy can improve the *ΔH*_*mix*_ predictions (as seen in Figure 7), this adjustment alone is not sufficient, as it impacts negatively the LLE predictions (Figure 6). This suggests that the residual term is not the main cause of the deviations observed on the LCST. Moreover, since hydrogen-bond parameters affect exclusively the residual (enthalpic) contribution, their adjustment cannot correct deficiencies in the combinatorial term. The fact that tuning the hydrogen-bond interactions improves the description of the enthalpy of mixing while failing to enhance the LCST and overall phase equilibrium suggests the existence of a trade-off in the balance of the Gibbs free energy.

Although the residual term accounts for energetic interactions, one may question whether its current formulation adequately captures dispersion forces in polymer–solvent systems. Variants of COSMO-SAC incorporating explicit dispersion contributions have been proposed, such as the COSMO-SAC-dsp model by Hsieh et al. (2014).^52^ However, recent comprehensive benchmarks on polymer systems, such as the work by Antolović et al. (2024),^38^ have demonstrated that this specific dispersion term has a negligible numerical effect on the phase equilibria of polymer solutions. While a reformulation of the dispersion contribution based on surface area fractions has been suggested as a potential pathway for improvement in their work, such a modification would require a fundamental change of the model.

Analyzing the different deviations observed for UCST and LCST from a thermodynamic perspective, UCST behavior is comparatively simpler, as increasing temperature progressively reduces nonideality and drives the system toward a single homogeneous phase. In contrast, the LCST in aqueous PEG corresponds to a low-temperature LCST associated with temperature-dependent changes in hydrogen bonding.^53^ However, the limited sensitivity of the predicted LCST to variations in hydrogen-bond energy indicates that the dominant source of deviation in the model does not arise solely from the residual enthalpic term, but rather from how enthalpic and combinatorial contributions balance to determine the curvature of the Gibbs free energy of mixing. Because this transition depends on such a subtle balance, small inaccuracies in this interplay may substantially affect LCST prediction.

### 5.4 Combinatorial Contribution Effect in the LLE

Figure 8 shows the influence of the combinatorial contribution and free volume effect on the prediction of PEG and water LLE. The full line represents the FH combinatorial contribution, in terms of COSMO volume, without any free volume contribution. It is possible to obtain a full closed loop, but with large deviations from the experimental data, as previously observed. As we used the differences between the molar volume of the compound and the COSMO volume, water becomes less soluble in the polymer, and both UCST and LCST start to increase, with a significant impact on LCST.

**Figure 8:**
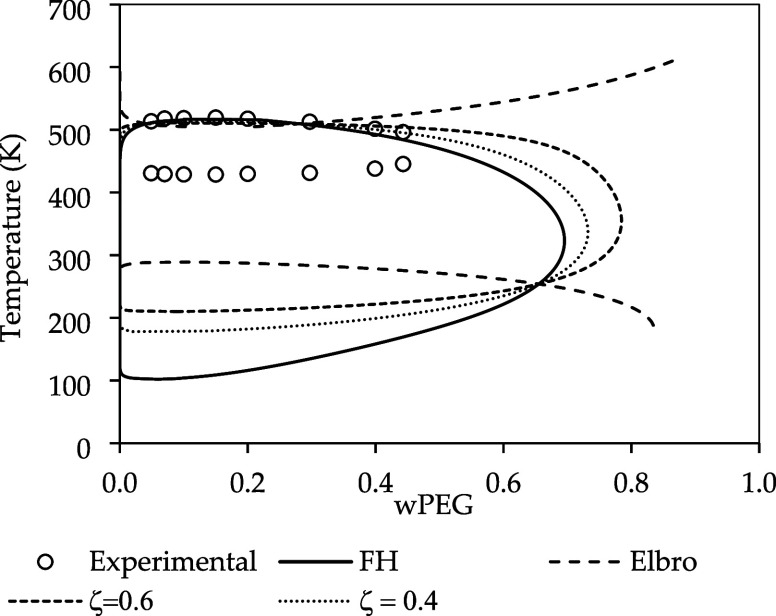
Influence of the volume parameter ζ in the LLE of PEG (3350 g·mol^–1^) and water; ζ = 0 corresponds to the Flory–Huggins^32^ (FH) limit, while ζ = 1 recovers the Elbro et al.^13^ formulation. Experimental data are taken from.^50^.

As we increase the molar volume even further, with higher values of *ζ*, a transition occurs when the molar volume exceeds COSMO volume, causing the closed loop to fade and resulting in a swap of positions between the UCST and LCST. This transition occurs near *ζ* = 1, at which point the combinatorial term turns into the Elbro et al.^13^ equation. Increasing *ζ* values even further, which means a greater free volume, the LLE remains a UCST and LCST and no closed loop is formed. A similar result was achieved by Kuo et al.,^12^ who used COSMO-SAC with Staverman–Guggenheim combinatorial term and obtained a UCST for the PEG/water system; however, when the Elbro et al.^13^ combinatorial term was used, they obtained just an LCST. Mathematically, this occurred due to the larger values of the combinatorial term when FV is included, surpassing the residual contribution. We also explored the impact of the exponent *p* in the *r*_*i*_ of FH, as shown in the work of Zhong et al.,^24^ but better results were only achieved using values above 1, which have no physical meaning. This indicates that error compensation was occurring to obtain such results.

Recently, Krooshof and de With^54^ observed that the FH term is the only combinatorial term, among those commonly used, that is physically consistent and correctly represents the combinatorial entropy. They demonstrated that shape-based models violate the Gibbs probability normalization condition, whereas free-volume formulations (e.g., Elbro) incorrectly introduce temperature-dependent energetic contributions into the combinatorial term, which should be strictly athermal. Additionally, Kouskoumvekaki et al.^55^ results showed that the Elbro equation yields good results for short-chain solutes in long-chain solvents, whereas its performance deteriorates in the opposite scenario, namely long-chain solutes in short-chain solvents, which was also emphasized by Grigorash et al.^56^ for COSMO-RS. This limitation is particularly relevant for aqueous polymer systems, such as PEG–water mixtures, in which the polymer necessarily behaves as the long-chain solute. These findings suggest that the main challenge in accurately modeling the LCST using COSMO-SAC may lie in the proper representation of the combinatorial contribution. For other activity coefficient models, Voutsas and Tassios^57^ demonstrated that UNIFAC also encounters significant limitations in highly size-asymmetric systems, particularly when a large solute is dissolved in a small polar solvent. Under these conditions, they reported larger deviations when the Elbro et al.^13^ combinatorial term was employed instead of the classical FH^32^ formulation.

In addition, Staudt et al.^11^ pointed out that the combinatorial term of COSMO-SAC probably requires improvement to achieve reliable LLE predictions for polymer–solvent systems. A direct comparison between model predictions and the experimental entropy of mixing is not feasible for the PEG–water system due to the lack of experimental polymer activity coefficients in the two-phase region, as later discussed in Section 5.6. Consequently, the present analysis is restricted to model-consistency considerations.

### 5.5 Conformation Influence in the LLE

Different conformers were used to assess their effect on the LLE. For this purpose, the structures obtained from the MD simulation of Silva et al.^15^ were used, which were kindly provided to us. In their work, MD simulations of PEG in water were performed to generate realistic conformers. The four specific structures selected by the authors were chosen based on a joint probability analysis of the Radius of Gyration and Solvent Accessible Surface Area (SASA) according to the methodology described by Zhou et al.^58^ This selection aimed to cover the most statistically probable conformations found in solution. These structures are shown in Figure 9.

**Figure 9:**
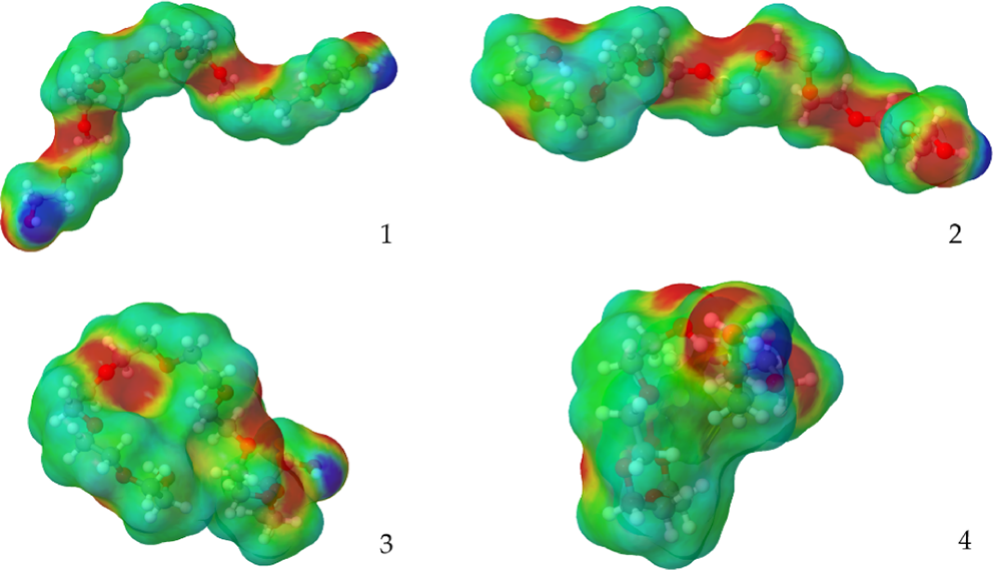
Representative molecular conformations (1–4) of PEG obtained from molecular dynamics simulations reported by Silva et al.^15^

When using TZVP and FH, all conformers converged to an LLE, and the results are presented in Figure 10. It is worth mentioning that conformers 3 and 4 were the most representative in the MD simulation. The use of ζ =1 (Elbro et al. combinatorial term) produced a UCST and LCST behavior for all conformers, and no closed loop was obtained. We also attempted to perform calculations using TZVPD-FINE, but no LLE was formed using FH, and with *ζ* = 1, just UCST and LCST were again found.

**Figure 10:**
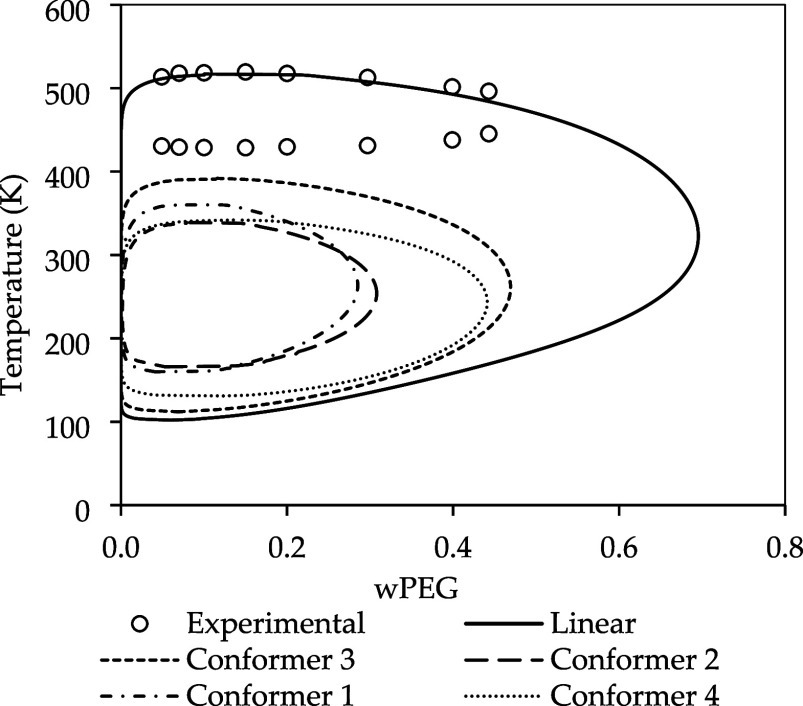
Influence of conformation in the predicted LLE of PEG (3350 g·mol^–1^) and water. Experimental data taken from Bae et al.^50^

As shown in Figure 10, when using conformer 3, the PEG-rich phase decreased its mass composition, allowing more water to become soluble in the polymer. When a conformer is used instead of the linear structure, the oxygens become hidden by other atoms, reducing the capability of water to solubilize. However, a much more neutral area is hidden than positive areas, which favors the solubility of water in PEG, since a larger neutral area makes it more difficult for water to dissolve due to less polarity. This can be observed in the σ-profile presented in Figure 11, where the conformer has a notably lower neutral area.

**Figure 11:**
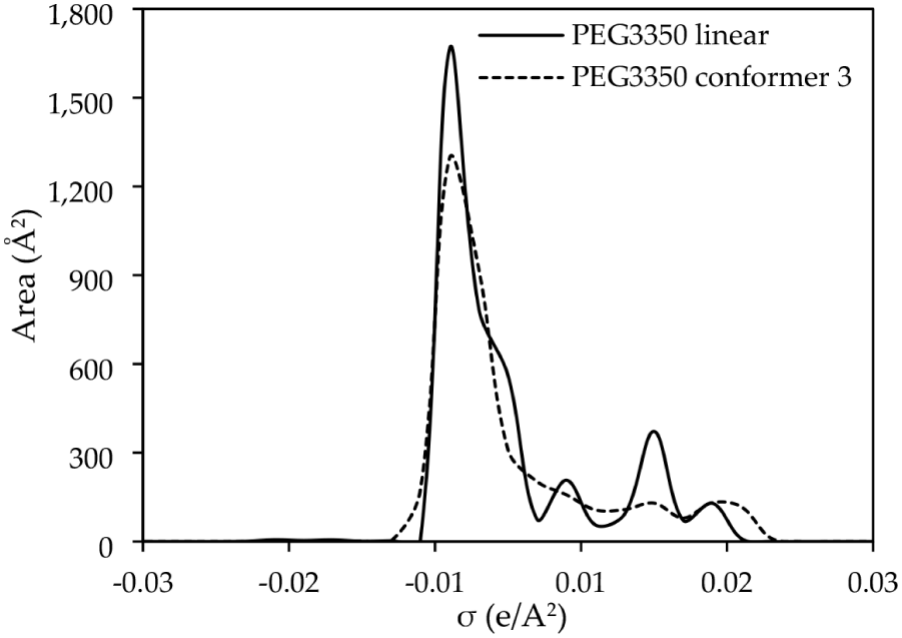
σ-profile comparison between the linear PEG and conformer 3, generated with JCOSMO.^59^

While with the conformer it was possible to describe the compositions in the rich phase with better agreement, the equilibrium temperature is poorly described, with a greater deviation observed for UCST, which is even more underestimated. A similar analysis could be done for the other conformers, where, in some cases, PEG is even less soluble in the PEG-rich phase.

Although the use of multiple polymer conformations obtained from MD is explored herein, the conformers are treated deterministically rather than statistically averaged. In principle, COSMO-based approaches may account for conformational ensembles by applying Boltzmann weighting based on quantum chemical energies. However, the current COSMO-SAC implementation in JCOSMO does not support Boltzmann averaging of multiple conformers within a single compound description. One possible strategy would be to treat different conformers as distinct pseudocomponents, with temperature-dependent populations determined from their relative quantum chemical energies. However, introducing such an approach would increase the dimensionality of the phase equilibrium problem, requiring a consistent treatment of their temperature-dependent populations and adding considerable computational complexity. While COSMO-RS implementations, such as those available in TURBOMOLE, allow the use of multiple conformers as a single component, they are not currently able to resolve the LLE of PEG–water systems due to numerical limitations arising from the large molar mass asymmetry between polymer and solvent, as discussed by Silva et al.^15^ Consequently, the conformer analysis presented here should be interpreted as a sensitivity analysis, aimed at assessing the influence of polymer conformation on LLE predictions.

### 5.6 Activity Coefficient and LLE Formation

This section aims to clarify the thermodynamic mechanism underlying the LLE predicted for the PEG–water system and to help identify the fundamental limitations of COSMO-SAC in describing polymer–solvent phase behavior. Unlike the parametric analyses presented in previous sections, the discussion here focuses on the activity coefficients and their role in LLE formation.

In classical thermodynamic interpretations, LLE occurs at high activity coefficients because this indicates that interactions between molecules of different components are less favorable than those between like molecules. This results in phase separation, as each component prefers to reside in a phase where intermolecular interactions are more favorable. However, in the PEG/water system, LLE formation occurs at very low activity coefficients. The water activity coefficient typically ranges from 0.2 to 1 in all temperature range, as calculated by Tritopoulou et al.^26^ from VLE experiments of Ninni et al.,^60^ Herskowitz and Gottlieb,^61^ Eliassi et al.,^62^ and Striolo and Prausnitz.^63^ In contrast, experimental PEG activity coefficients cannot be calculated using VLE data due to PEG’s negligible vapor phase composition. LLE data cannot provide these coefficients, as there are insufficient variables to solve for them. SLE data offers a way to calculate PEG activity coefficients, but only at low temperatures, limiting its applicability. Silva et al.^15^ calculated the PEG activity coefficient at 298 K from SLE data, finding very low activity coefficient values. As PEG’s molar mass increases, the activity coefficient becomes even lower, reaching values on the order of 10^–161^ at 35,000 g/mol. Additionally, one consequence of this limitation is that experimental excess entropy cannot be evaluated under LLE conditions using formal thermodynamic relations, such as *s* ^*E*^ = (*h^E^* – *g* ^*E*^)/*T*, because this requires accurate activity coefficients for both components.

One might expect that increasing temperature would raise these activity coefficients, eventually leading to LLE formation. However, this is not observed; COSMO-SAC predicts that PEG activity coefficients remain very low even at higher temperatures, where LLE occurs. UNIFAC-FV in the work of Tritopoulou et al.^26^ also predicts negative excess Gibbs energy across all temperature ranges, implying negative logarithms of PEG activity coefficients, while the logarithm of the water activity coefficient remains near zero or slightly negative. This highly negative ln­(γ) for PEG, and the large disparity in activity coefficients between PEG and water, suggests that one component may prefer one phase over the other much more strongly than the other component, which could lead to a phase separation.

Figure 12 shows the formation of the LLE, mathematically, with the activity coefficients predicted by COSMO-SAC. The *y*-axis in Figure 12 is the sum of the logarithm of the activity coefficient of the component and the logarithm of the composition, while the *x*-axis is the mass fraction of PEG. Given that the fugacity of each phase must be equal, the classic liquid–liquid equilibrium criteria in terms of activity coefficients can be obtained:^12^

15 $$x_{i}^{\alpha }\gamma_{i}^{\alpha }=x_{i}^{\beta }\gamma_{i}^{\beta }$$

if the natural logarithm is taken from both sides of the equation, it is possible to obtain that

16 $$\ln x_{i}^{\alpha }+\ln\gamma_{i}^{\alpha }=\ln x_{i}^{\beta }+\ln\gamma_{i}^{\beta }$$

**Figure 12:**
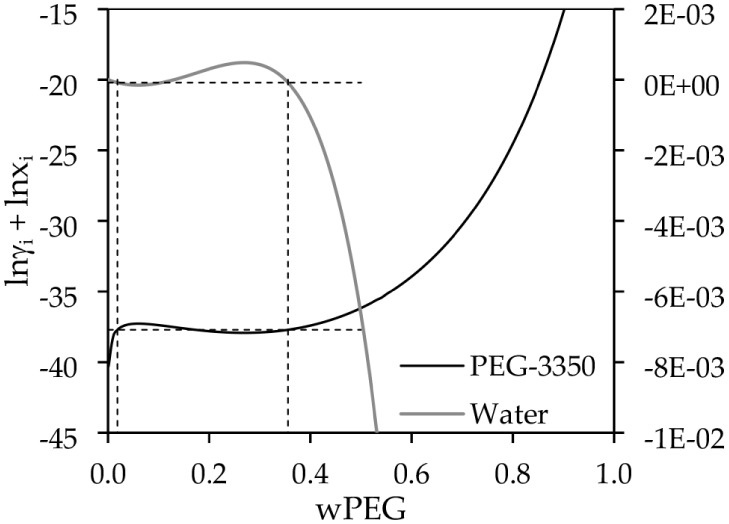
PEG (3350 g·mol^–1^) and water LLE formation at 480 K. The left and right *y*-axes correspond to ln­(γ_i_) + ln­(x_i_) for PEG and water, respectively.

Thus, the sum of the logarithms must be equal in both phases, as can be observed in Figure 12. The dashed lines correspond to guidelines that follow the equilibrium compositions found by the model. As it is possible to observe, the LLE was found even with both components having very low activity coefficients, below the unit. This method was chosen to observe the LLE formation because common graphical methods fail due to the large disparity between the activity coefficients of both components.

In summary, Figure 12 shows that the model predicts LLE formation through a large asymmetry in activity coefficients, with lnγ_PEG_ being highly negative while lnγ_water_ remains near zero. This asymmetry suggests that the system creates a preferential partitioning where one component favors one phase over the other, mathematically satisfying the equilibrium criterion and leading to phase separation even without the typical condition of γ_i_ ≫ 1. However, whether this mathematical prediction correctly represents the actual thermodynamic driving forces in the PEG-water system remains uncertain, given the considerable quantitative deviations observed in LCST predictions and the complex interplay between enthalpic and entropic contributions in this system.

### 5.7 Influence of PEG Molar Mass in LLE

Figure 13 shows the impact of the molar mass in the LLE predicted by the model. The predicted UCST increases with molar mass, as expected, but still underestimates the temperature. The predicted LCST almost does not change with molar mass, which is not observed in the experimental data. These results suggest that the COSMO-SAC model still needs improvements to capture the effects of molar mass increase, but possibly a correction in the model, especially in the combinatorial term, that could fix the LCST high underestimation, may also improve the description of the LLE variation with molar mass.

**Figure 13:**
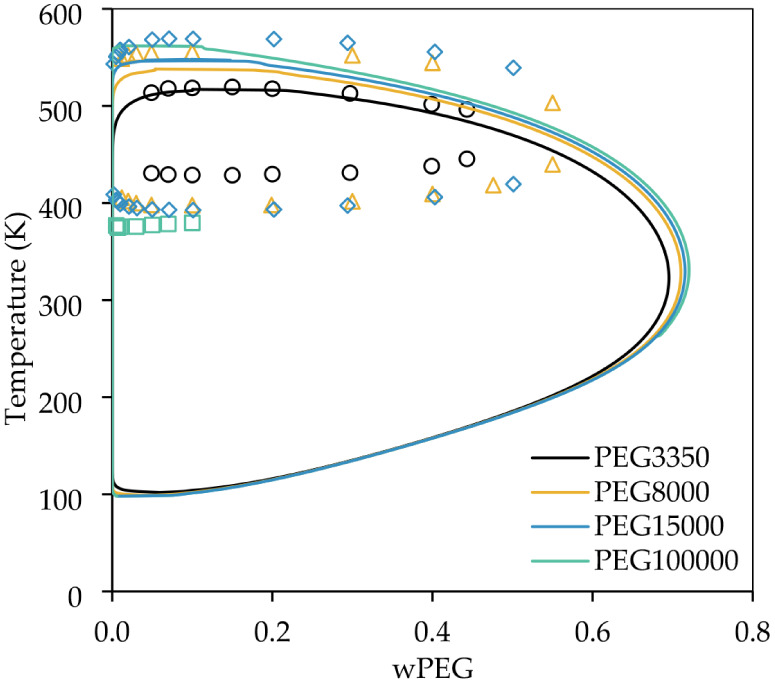
Influence of PEG molar mass in LLE. Experimental data from ref 50.

Furthermore, beyond the specific contributions of the residual and combinatorial terms, the deviations in UCST and LCST observed in Figure 13 can also be attributed to the theoretical limits of the model itself. COSMO-SAC, like most activity coefficient models used in engineering (e.g., UNIFAC), operates within a mean-field approximation. This implies that the model neglects the long-range density and concentration fluctuations that dominate system behavior near critical points. Consequently, mean-field theories typically predict a parabolic coexistence curve rather than the flatter shape observed experimentally in polymer solutions near the critical points. The absence of crossover corrections or renormalization group theory in the current framework means that quantitative deviations near the UCST and LCST are theoretically expected. Therefore, the observed errors in UCST and LCST prediction should be interpreted as a cumulative effect of the combinatorial and residual terms of the model and the inherent inability of the mean-field approximation to capture critical fluctuation physics.

It is also possible to observe in Figure 13 that the proposed algorithm could predict the separation in the entire data range, even when considering high molar masses. However, due to the model predict very low compositions in the poor PEG phase, even in terms of mass fractions, there is a limit of molar mass increase that can be used, in virtue of numerical limitations. Despite that, the algorithm proposed can be used in most applications, and if the model achieves a better description of experimental data, with future improvements, this numerical problem would also be solved.

## 6 Conclusions

A strategy for constructing σ-profiles of polymers was proposed, adapting the classical procedure to explicitly account for end groups, regardless of the polymer’s molar mass. With this improved procedure, it was possible to obtain LLE predictions closer to those obtained using the full PEG molecule, thereby reducing inconsistencies.

Additionally, an algorithm was developed to solve the LLE of polymers, which remains a challenge for classical thermodynamic software due to the large disparity in molar mass between the polymer and the solvent. The proposed method successfully resolved the PEG-water phase equilibrium and constructed the LLE diagram without requiring any model derivatives, while overcoming the numerical limitations.

For the first time, the closed-loop LLE behavior of PEG and water was predicted using COSMO-SAC without fitting any binary interaction parameters and relying solely on universal parameters. The model performed well in predicting the UCST and the high-temperature region of the experimental data. However, the LCST was poorly predicted, showing substantial deviations from experimental values.

The residual contribution of COSMO-SAC showed limitations in predicting the enthalpy of mixing, and adjusting the hydrogen bond energy parameters was not enough, as it deteriorated the LLE predictions while improving Δ*H*_*mix*_. This demonstrates that the large deviations observed cannot be attributed to an incorrect temperature dependence of hydrogen bonding alone. The combinatorial term appears to be the main challenge in accurately modeling the LCST, which also influences the type of the LLE diagram formed. Overall, the dominant source of error is an incorrect balance between the residual and combinatorial contributions, rather than a failure of either term in isolation. A combinatorial term inspired by PC-SAFT may be a solution for polymer–solvent systems where the free volume plays a significant role, but improvements to the residual term, perhaps by turning HB energy composition-dependent, also seem necessary for a precise description of PEG-water systems.

## Supplementary Material



## Notes

Section S1: reference oligomer; Section S2. determination of volume and surface parameters; Section S3: JCOSMO .custom file example; Figure S1: Example of the JCOSMO .custom file used in this work for the construction of the PEG σ-profile (PDF)
