# Supplementary material for: Unveiling the Constraints of COSMO-SAC for PEG-Water Liquid–Liquid Equilibrium Prediction
Source: Ind Eng Chem Res. 2026 May 5;65(19):10175–86. doi: 10.1021/acs.iecr.5c04482 (PMC13206633; doi:10.1021/acs.iecr.5c04482)
Supplement: Supplementary file 1 [file ie5c04482_si_001.pdf]

Supporting Information

Unveiling the Constraints of COSMO-SAC for PEG-Water Liquid-Liquid Equilibrium Prediction

Edgar T. de Souza Jr.[a], Murilo L. Alcantara[b], Paula B. Staudt[a], João A. P. Coutinho[b]*, Rafael de P. Soares[a]*

[a] *Virtual Laboratory for Properties Prediction (LVPP), Chemical Engineering Department, Federal University of Rio Grande do Sul, Rua Ramiro Barcelos, 2777, CEP 90035-007, Porto Alegre, RS, Brazil.*

[b]*CICECO - Aveiro Institute of Materials, Chemistry Department, University of Aveiro, Aveiro, Portugal*

*jcoutinho@ua.pt, *rafael.pelegrini@ufrgs.br

*Corresponding author at: CICECO - Aveiro Institute of Materials, Chemistry Department, University of Aveiro, Aveiro, Portugal
*Corresponding author at: Virtual Laboratory for Properties Prediction (LVPP), Chemical Engineering Department, Federal University of Rio Grande do Sul, Rua Ramiro Barcelos, 2777, CEP 90035-007, Porto Alegre, RS, Brazil.
Email addresses: jcoutinho@ua.pt (João A. P. Coutinho), rafael.pelegrini@ufrgs.br (Rafael de P. Soares).
Tel.: +351 234 370 200 (João A. P. Coutinho), +55 (51) 3308 2854 (Rafael de P. Soares).

This Supplementary Material provides an illustrative example of the JCOSMO .custom file employed in this work to construct the σ-profile of polyethylene glycol (PEG) with a target average molar mass, following the methodology described in Section 3 of the main manuscript.

S1. Reference oligomer

The COSMO surface is generated from a PEG oligomer previously optimized and calculated at the selected level of theory. The corresponding .cosmo file serves as the reference structure for the defining terminal groups and the repeating unit.

S2. Determination of volume and surface parameters

The repeating unit volume and surface area were obtained from COSMO calculations of PEG oligomers, all containing an odd number of repeating units. The repeating unit parameters are calculated from the difference in total COSMO volume and surface area between the two largest consecutive oligomers.

S3. JCOSMO .custom file example

The JCOSMO .custom file is a plain text file used to define weighted COSMO surface segments for polymer σ-profile construction, and it is presented in Figure S1. Each command specifies how information from the reference oligomer is used during σ-profile generation. The LOAD command specifies the reference PEG oligomer .cosmo file from which the COSMO surface information is retrieved. The VOLUME command defines the total molecular volume of the target polymer, expressed in $Å^3$, and is consistent with the target average molar mass employed in the COSMO-SAC calculations. The ONLY_FOR command identifies the atom indices of the reference oligomer whose COSMO surface segments are retained in the σ-profile construction. The ATOM_MULTIPLIER command assigns multiplicative weights to the COSMO surface segments associated with each selected atom. Atoms belonging to the PEG terminal groups are assigned to a unit multiplier, whereas atoms corresponding to the repeating unit are assigned to a multiplier equal to the number of basic units N_b, as defined by Eq. (8) in the main manuscript:

$$N_b = \frac{MW_p - \sum MW_e}{MW_r}$$

Text fragments preceded by the symbol # are treated as comments and are ignored by JCOSMO during file parsing. They are included solely to improve readability and to indicate the assignment of terminal groups and repeating unit atoms within the file.

Figure S1. Example of the JCOSMO .custom file used in this work for the construction of the PEG σ-profile.

```
LOAD PEG_414

VOLUME 4051.58538 #Volume of the full polymer [Å³]

ONLY_FOR        1 2 3 26 27 28 29 30     13 14 44 43 15 45 46     59 60 61 62 63 64 65 66

ATOM_MULTIPLIER  1 1 1 1 1 1 1 1         Nb Nb Nb Nb Nb Nb Nb      1 1 1 1 1 1 1 1
```
